# Proceedings of Societies

**Published:** 1884-05

**Authors:** 


### PROCEEDINGS.

### Proceedings of Societies.

The Tennessee Dental Association held its Eighteenth Annual Session at Nashville, in the Lecture Hall of the Department of Dentistry of Vanderbilt University, the 19th, 20th, and 21st of February.

The following officers and members were present:

Dr. J. Y. Crawford, President, Nashville.

Dr. J. U. Lee, Vice-President, Chattanooga.

Dr. D. R. Stubblefield, Second Vice-President, Nashville.

Dr. D. B. Blakemore, Recording Secretary, Nashville.

Dr. R. P. Jones, Corresponding Secretary, Columbia.

Dr. W. H. Morgan, Treasurer, Nashville.

Drs. H. E. Beach and H. M. Acree, Clarkesville; A. F. Claywell, Lebanon; J. H. Prewitt, Madisonville, Ky.; P. D. Houston, Lewisburg; E. G. Scott, Culleoka; W. B. Spencer,

Jackson; W. W. Baum, M. W. Williams, and G. E. Medley, Hopkinsville, Ky; R. A. B. Moyers, Jasper; T. G. Holder, Gallatin; W. F. Fowler, R. Russell, R. R. Freeman, G. B. Clements, R. M. Boyle, J. C. Ross, Gordon White, S. J. Cobb, R. B. Lees, Henry W. Morgan, and A. T. Kline, of Nashville.

Among the many visitors were noticed Dr. Wm. R. McWilliams, of Athens, Ala., President of State Board of Dental Examiners; Prof. Ambrose Morrison, Drs. T. O. Summers and J. M. Coyler, of Nashville; B. G. Blackwell, of Hempstead, Texas; A. D. Lampkin, of Pulaski; J. T. Taylor, of Brownsville; R. H. McNair, of Woodville, Miss.; T. C. West, of Fayette, Miss.; J. M. Clark, of Mt. Hope, Ala., and O. F. Gambati, of Albany, Ga.

The Association was called to order by President Crawford, and opened with prayer by B. G. Blackwell, of Texas.

Prof. R. Russell delivered an address of welcome, and the President read the annual address of that officer, considering the "Development of our Profession."

The Executive Committee had furnished in the invitations the following topics for essays and discussion, and had also invited members and non-member visitors to prepare papers on any of them, or any other subject likely to entertain and instruct:

1. Artificial Crowns.
2. Art in Mechanical Dentistry.
3. Pulpless Teeth, Alveolar Abscess (acute and chronic treatment of).
4. Carious Molars and Bicuspids.
5. Anæsthetics.
6. Pulpitis and Periostitis.
7. Pyorrhea Alveolaris.
8. Treatment of Irregularities.
9. Incidents of Office Practice.

All the topics received a liberal discussion, and papers were read upon the following by the gentleman named:

"Art in Mechanical Dentistry," Dr. P. D Houston; "Treatment of Pulpless Teeth," Dr. W. H. Morgan; "Carious Molars and Bicuspids," Dr. M. W. Williams; "Anæsthetics,"* Dr. R. R. Freeman; "Pyorrhea Alveolaris," Dr. Henry W. Morgan; "Incidents of Office Practice," Dr. W. F. Fowler; "History of Dentistry," Dr. J. U. Lee; "Reflex Nerve Action," Dr. G. A. McPeters, Pulaski. (Not present; paper read by Dr. Freeman).

The discussions and papers were much better than the Association had ever enjoyed before. Throughout, the meeting was characterized by the greatest harmony and good feeling. It was generally conceded to be the most successful meeting this Association had ever held.

A letter from Dr. B. H. Catchings, of Atlanta, Ga., was read asking the aid of the Association in the organization of a "National Dental Association," to hold its meetings annually at Washington (with a Museum and Library to be located there) by the election of six delegates; said National Association to be made up of six delegates from each of the States of the Union. The communication was referred to a committee, which returned it without any recommendation. After some consideration the matter was laid on the table.

Tennessee has no law regulating the practice of Dentistry, though almost every legislative body since 1867 has been asked to pass one. The committee having in charge this matter was continued.

The officers elected at this meeting were:

President—J. U. Lee, Chattanooga.

First Vice-President—Henry W. Morgan, Nashville.

Second Vice-President—W. F. Fowler, Nashville.

Recording Secretary—D. B. Blakemore, Nashville.

Corresponding Secretary—R. B. Lees, Nashville.

Treasurer—W. H. Morgan, Nashville.

The Association will meet in Nashville the third Tuesday in February, 1885.

H. W. M.

---

*This subject probably elicited more debate and interest than any other, and the expression for and against the use of these agents was quite full, the prevailing opinion being that they should be resorted to only in extreme cases, and that chloroform should never be administered for the extraction of teeth.

